# Dynamic Changes in Coronary Flow Pattern During Transcatheter Aortic Valve Replacement in Severe Aortic Stenosis

**DOI:** 10.1016/j.jaccas.2021.07.005

**Published:** 2021-10-06

**Authors:** Wataru Suzuki, Yusuke Nakano, Hirohiko Ando, Hiroaki Takashima, Tetsuya Amano

**Affiliations:** Department of Cardiology, Aichi Medical University, Nagakute, Japan

**Keywords:** Aortic stenosis, aortic valve, coronary flow pattern, systolic coronary flow reversal, transcatheter aortic valve replacement, transesophageal echocardiography, AS, aortic stenosis, LAD, left anterior descending artery, TAVR, transcatheter aortic valve replacement, TEE, transesophageal echocardiography

## Abstract

Coronary flow reserve in patients with severe aortic stenosis decreases even in the absence of coronary stenosis. In this case, the dynamic changes in the coronary flow pattern around transcatheter aortic valve replacement were observed by periprocedural transesophageal echocardiography. (**Level of Difficulty: Intermediate.**)

## Central Illustration

**Figure:**
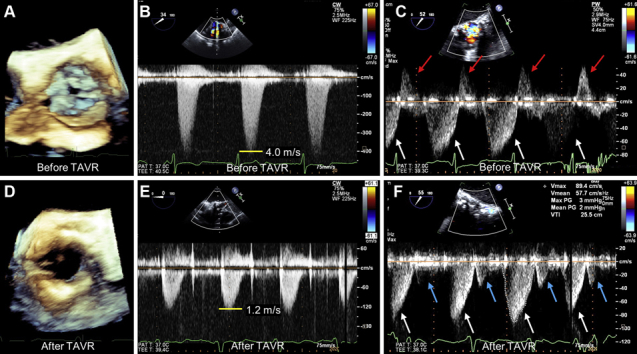

The coronary flow pattern in the left anterior descending artery (LAD) generally shows a biphasic forward flow, consisting of a small systolic and a large diastolic flow. In patients with aortic stenosis (AS), coronary flow reserve decreases even in the absence of coronary stenosis. This is related to the severity of AS, increased hemodynamic load, and reduced diastolic perfusion time (1). Transesophageal echocardiography (TEE) allows a detailed proximal LAD flow pattern assessment (Supplemental Figure 1).

**Figure 1:**
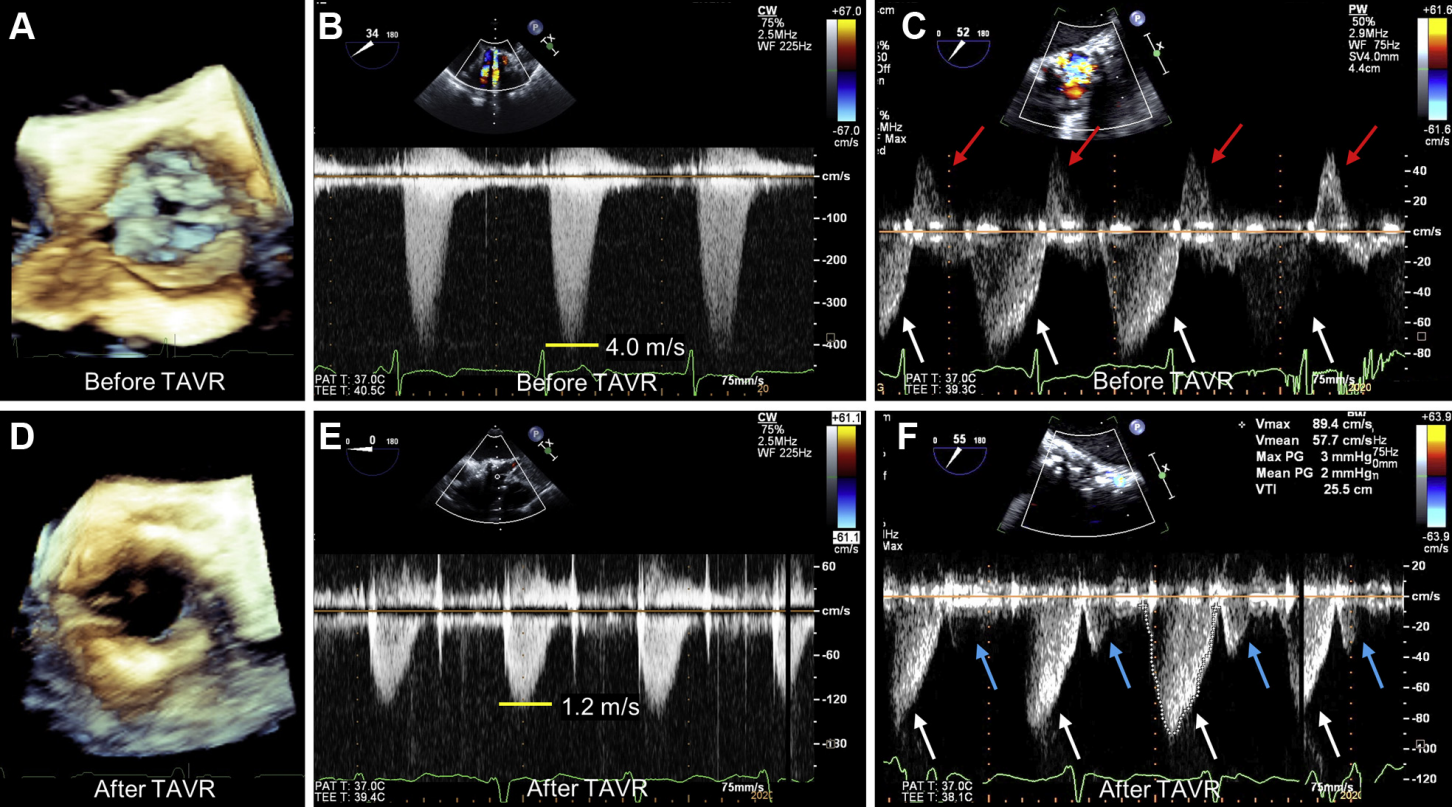
Transesophageal Echocardiography Before and After TAVR **(A)** Severely restricted aortic cusp motion. **(B)** Aortic valve peak velocity of 4.0 m/s before transcatheter aortic valve replacement (TAVR). **(C)** Coronary flow pattern before TAVR: reversed coronary flow during systole **(red arrow)** and forward coronary flow during diastole **(white arrow)**. **(D)** Aortic valve complex after TAVR. **(E)** Aortic valve peak velocity of 1.2 m/s after TAVR. **(F)** Coronary flow pattern after TAVR: biphasic forward flow with small systolic **(blue arrow)** and large diastolic flow **(white arrow)**.

An 85-year-old woman with severe AS was hospitalized to undergo transcatheter aortic valve replacement (TAVR) by the transfemoral approach. Her blood pressure was 108/71 mm Hg, and her pulse was 93 beats/min. Physical examination revealed a grade 3/6 systolic murmur over the upper sternal border. Preprocedural computed tomography showed a severely calcified aortic valve without coronary stenosis (Supplemental Figure 2). Preprocedural TEE showed preserved left ventricular contraction without aortic regurgitation. Aortic cusp motion was severely restricted (Figure 1A, Video 1), and the aortic valve area was 0.49 cm^2^. The aortic valve peak velocity, assessed from a deep transgastric long-axis view, was 4.0 m/s (Figure 1B). Coronary flow in the proximal LAD was evaluated by pulsed-wave Doppler of TEE at the position of the upper esophagus, which showed systolic flow reversal and a forward diastolic flow before TAVR (Figure 1C). Following facility standards, a 23-mm Sapien 3 (Edwards Lifesciences) was successfully implanted without complications, including postprocedural aortic regurgitation (Figure 1D, Video 2). Postprocedural TEE revealed the aortic valve peak velocity reduced to 1.2 m/s (Figure 1E). TEE-based LAD flow measurement just after TAVR showed a normal biphasic forward flow pattern with a small systolic and a large diastolic flow; the coronary flow during systole had changed from reverse to forward (Figure 1F). The patient had an uneventful course until 5 months after TAVR.

In this case, measurements by TEE revealed dynamic changes in coronary flow patterns just after TAVR. The systolic flow reversal due to AS can be explained by: 1) the increased intraventricular pressure, which pushes the blood back from the intramyocardial vessels into the epicardial coronary artery during systole; and 2) the decreased pressure of the aortic sinus caused by the Venturi effect, which reverses blood flow from the proximal LAD back into the left main trunk. A previous report has described normalization of the flow reversal pattern after surgical aortic valve replacement (2). However, to our knowledge, this is the first report showing the dynamic changes in the coronary flow pattern after TAVR.

## Funding Support and Author Disclosures

The authors have reported that they have no relationships relevant to the contents of this paper to disclose.

## Appendix

Supplemental Video 1 Three-dimensional echocardiography before TAVR

Supplemental Video 2 Three-dimensional echocardiography after TAVR
