# Supplementary material for: Dynamic Changes in Coronary Flow Pattern During Transcatheter Aortic Valve Replacement in Severe Aortic Stenosis
Source: JACC Case Rep. 2021 Oct 6;3(13):1480–2. doi: 10.1016/j.jaccas.2021.07.005 (PMC8511413; doi:10.1016/j.jaccas.2021.07.005)
Supplement: Supplemental Figures 1 and 2 [file mmc3.docx]

**Supplemental Figure 1. Illustration of pulsed-wave Doppler with transesophageal echocardiography**

**
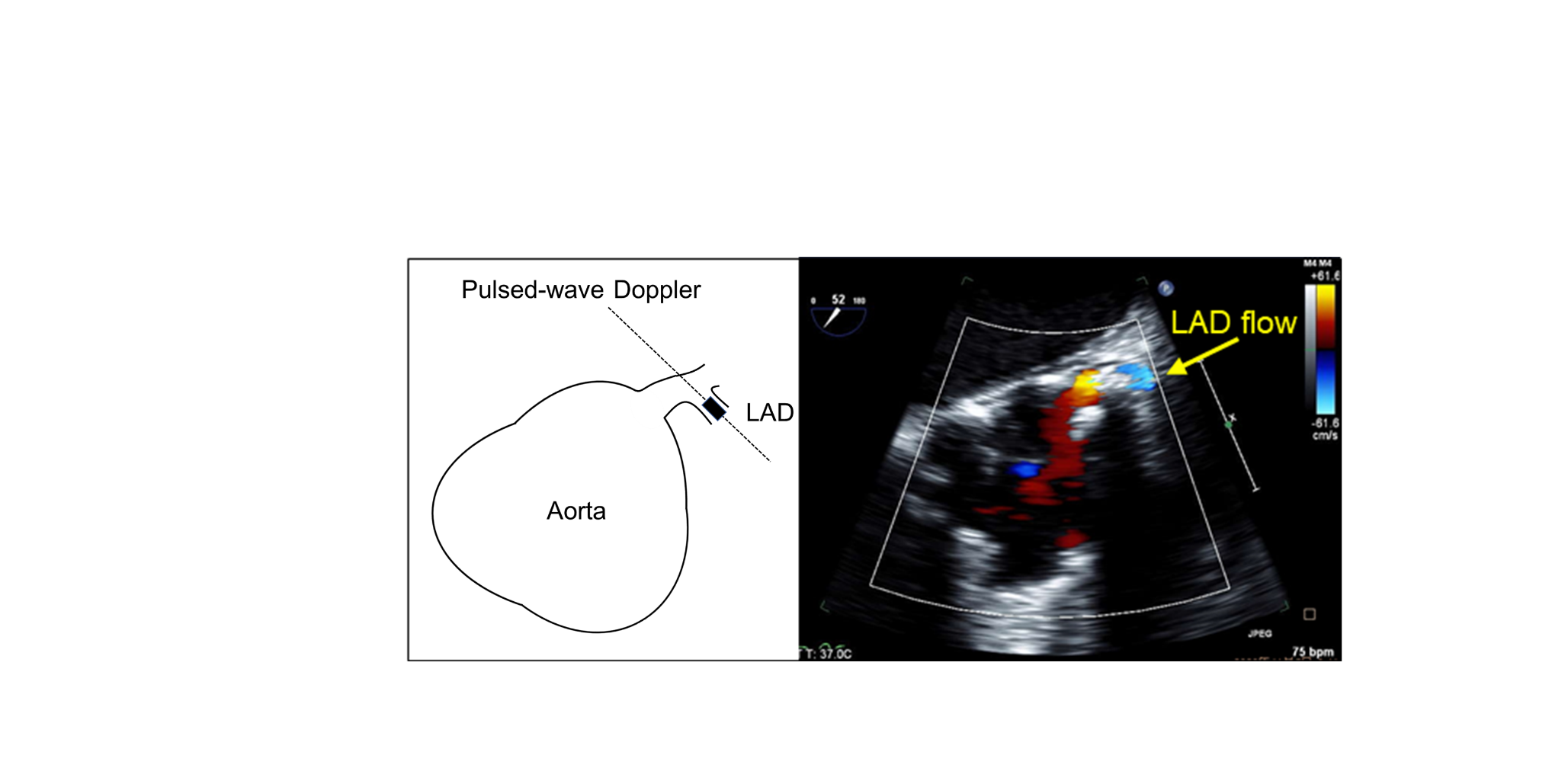
**

Position of pulsed-wave Doppler with transesophageal echocardiography to assess coronary flow. The transducer was positioned in the upper esophagus such that the left main coronary artery and the left anterior descending artery (LAD) could be assessed. The sample volume is placed over the proximal LAD (yellow-arrow).

**Supplemental Figure 2. Preprocedural computed tomography imaging**


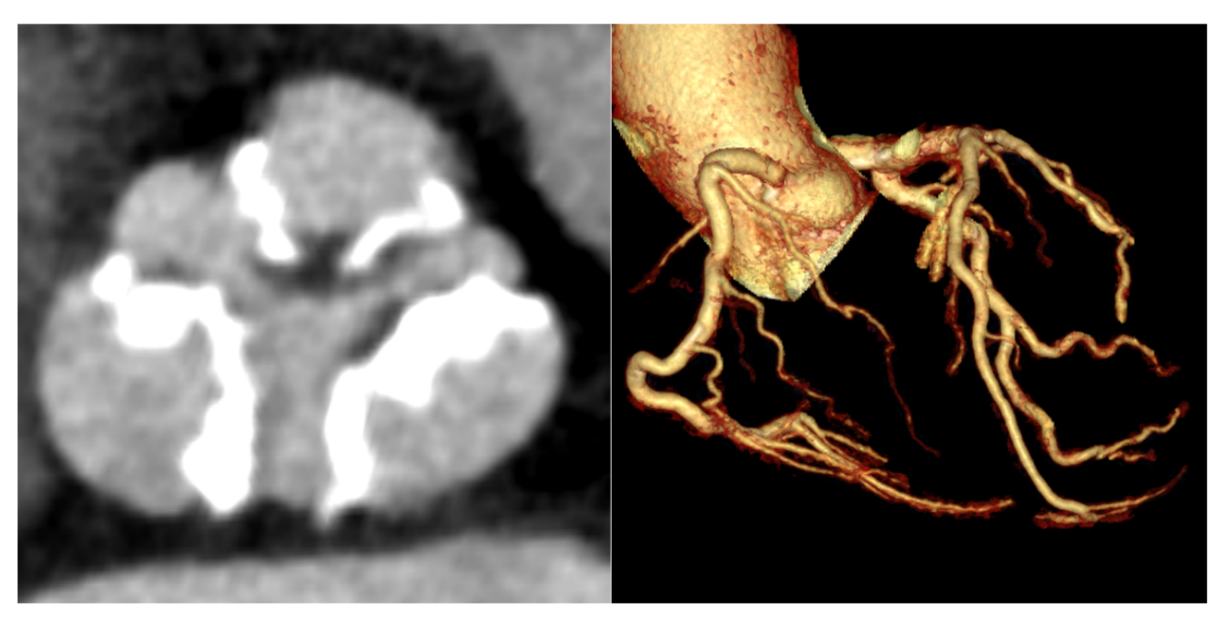


Preprocedural computed tomography showing severely calcified aortic valve and no coronary stenosis.
